# Home-based voluntary HIV counselling and testing found highly acceptable and to reduce inequalities

**DOI:** 10.1186/1471-2458-10-347

**Published:** 2010-06-17

**Authors:** Wilbroad Mutale, Charles Michelo, Marte Jürgensen, Knut Fylkesnes

**Affiliations:** 1 Department of Community Medicine, School of Medicine, University of Zambia, Lusaka, Zambia; 2 Centre for International Health, University of Bergen, Norway

## Abstract

**Background:**

Low uptake of voluntary HIV counselling and testing (VCT) in sub-Saharan Africa is raising acceptability concerns which might be associated with ways by which it is offered. We investigated the acceptability of home-based delivery of counselling and HIV testing in urban and rural populations in Zambia where VCT has been offered mostly from local clinics.

**Methods:**

A population-based HIV survey was conducted in selected communities in 2003 (n = 5035). All participants stating willingness to be HIV tested were offered VCT at home and all counselling was conducted in the participants' homes. In the urban area post-test counselling and giving of results were done the following day whereas in rural areas this could take 1-3 weeks.

**Results:**

Of those who indicated willingness to be HIV tested, 76.1% (95%CI 74.9-77.2) were counselled and received the test result. Overall, there was an increase in the proportion ever HIV tested from 18% before provision of home-based VCT to 38% after. The highest increase was in rural areas; among young rural men aged 15-24 years up from 14% to 42% vs. for urban men from 17% to 37%. Test rates by educational attainment changed from being positively associated to be evenly distributed after home-based VCT.

**Conclusions:**

A high uptake was achieved by delivering HIV counselling and testing at home. The highest uptakes were seen in rural areas, in young people and groups with low educational attainment, resulting in substantial reductions in existing inequalities in accessing VCT services.

## Background

Voluntary HIV counselling and testing (VCT) has strongly been promoted as essential in reaching universal access to HIV prevention, care, support and treatment, and the services have been scaled up in many low- and middle-income countries. However, access and uptake is still considered to be very low, and where VCT is readily available demands have often been surprisingly low [1-7]. The striking gap between what people say they would like to do and what they actually do when services are offered is indicating that the way the services are provided has a low acceptability in the population. It has also been shown that in many settings uptake of VCT has been positively correlated with factors such as male gender, higher educational attainment, and urban residence [1-3,8-10]. Such differences in use of HIV testing and counselling might be indicative of inequalities in access. However, reasons for differential use is poorly understood [7]. Numerous studies have concluded that there are serious barriers to use which are related to the way services are offered, particularly indicated by the disappointingly low acceptability of facility-based testing [11-13]. Barriers of this kind are likely to be socially patterned, and investigating inequalities in use raises important methodological considerations. Measuring acceptability will be critical in this regard, and the most valid methods will be through actually offering VCT to assess its acceptability [3,11].

The low demand of VCT calls for innovate ways of offering VCT [7,14]. Several alternative service designs for VCT have been explored, such as workplace VCT [13], mobile VCT [15] and home-based VCT [7,11,12,16-19], with substantially increases in acceptability compared to regular clinic-based VCT. However, there has been a strong movement within the international AIDS community to shift from voluntary to routine testing. Routine testing is now recommended for all individuals attending any health facility in countries with generalized epidemics, and evidence of some increase in the proportion ever tested has been documented [7,20,21]. However, routine testing seems to be based on the belief that testing is the key tool for HIV prevention, and concerns have been raised from a preventive perspective due to the low emphasis being placed on counseling for risk reduction [22].

Almost 25 years after the first case of AIDS was reported in Zambia, the country still faces severe epidemics. The HIV prevalence is estimated to be bout 15% among 15-49 year olds [23], but great geographical differentials in magnitude and trends have been revealed [24]. However, there is evidence of overall declines in HIV prevalence being associated with reduction in risk behaviours among young people. These declines are largely being associated with educational attainment [25,26]. Despite rapidly improving availability of VCT in Zambia, the proportion reporting being tested for HIV is still low [27,28]. A randomised trial on the acceptability of VCT revealed home-based VCT to be highly acceptable in an urban setting [12]. We offered home-based VCT to all participants in a population-based HIV survey conducted in selected urban and rural areas, and we investigated the intention of being tested for HIV, acceptability and to what extent home-based VCT affected inequalities in HIV test rates in rural and urban settings.

## Methods

### The population survey

The data stem from a population-based survey conducted in Zambia in 2003, and details of participation rates and detailed overall methodology have been reported elsewhere [29]. Similar population-based surveys were conducted in the same areas in 1995 and 1999 [12]. The survey employed stratified random cluster sampling of selected communities in selected urban and rural areas of Lusaka and Kapiri Mposhi districts respectively. Ten clusters in each district were selected using 'probability proportional to size. All household members aged 15-49 years who lived in the selected clusters were invited to participate in the study. The number of participants was 5035. The survey used structured questionnaires and face-to-face interviews to collect information from the participants on socio-demographic factors, health and sexual behaviour. All interviews were conducted at the household level.

### Voluntary counselling and testing

At the end of the interview being conducted as part of the population-based survey participants were asked if they were willing to have HIV testing arranged for them at their home or at any convenient place. All who expressed willingness (intention) to test for HIV were then followed up by trained counsellors who were part of the study, and two senior counsellors acted as supervisors during the period of service provision. The counsellors visited consenting participants (those expressing willingness) at home for pre-test counselling shortly after the interview, i.e. at the same day or the following day. When participants gave their consent, blood for HIV testing was collected and taken to the nearest VCT laboratory for testing. In the urban area post-test counselling and HIV results were offered the following day at home, whereas in rural areas this process could take longer and often 1-2 weeks due to long distances. More than 90% of those accepting VCT preferred to be counselled and to receive the result at home, and only a few preferred to receive the services at the local VCT centre. It was essential to maintain confidentiality at all times during the counselling sessions. The counsellors reported challenges in some households in terms of finding a convenient place where privacy could be secured.

All HIV testing was carried out at the local clinic using the same testing strategies as the national guidelines for VCT. BIONOR HIV-1 & 2 (BIONOR AS, Skien, Norway) paramagnetic particle assay was used as the first test. All reactive samples were tested again using a rapid test Capillus HIV-1/HIV-2 (Cambridge Biotechnology, Galway, Ireland). Services were offered free of charge, but no particular strategy was instituted in terms of long-term follow-up services to HIV-infected individuals other than providing information about existing support and care opportunities. The counsellors recorded outcome information (persons being tested and received the result). This information was then added (as a new variable) to the data from the population-based survey.

### Analytical strategy

Intercooled Stata version 8 for windows (Stata Corporation, College Station, Texas, USA) was used for data analysis. All tests for statistical significance took into account the sampling design effect by using the survey data analysis function in Stata.

We employed five measures in the analyses: 1) "Intention" (or willingness) was measured based on the question "Would you like us to arrange for you to be HIV tested?"; 2) "Before" was defined as the proportion reporting to have ever been tested for HIV before offering home-based VCT and is thus equal past exposure to testing as measured in the survey (ever tested); 3) "Uptake" was measured as the proportion tested and receiving the result as a result of offering home-based VCT; 4) "Acceptability" was defined as the proportion of individuals who intended to be tested and received their results [3]; 5) "After" was measured as the proportion of all survey participants ever tested after having being offered home-based VCT, i.e. exposure to testing among all participants in the population-based survey after the home-based VCT intervention, i.e. calculated by updating the survey data with the data on uptake. Logistic regression was used to test differences between groups and changes in the distribution of exposures to HIV testing by selected socio-demographic characteristics (age, sex, marital status, residence, educational attainment) comparing the situation before and after offering home-based VCT.

### Ethics

The survey protocol received clearance from the University of Zambia Research and Ethics Committee. Participation in the study was based on written consent.

## Results

Table 1 gives an overview of the past experience with HIV testing (before), intention to be tested and the decisions to use the home-based services.

**Table 1:** Overview of HIV testing history, testing intentions and decisions related to actual use of home-based VCT.

Previously tested N = 895			Not Previously tested N = 4117		
**Not accept VCT**	**Accept VCT**		**Not accept VCT**	**Accept VCT**	

	**Did not use**	**Did use**		**Did not use**	**Did use**

549^1^	85	251	2799^2^	293	957

^1 ^10 individuals in this group did use VCT (excluded from in this table)

^2 ^68 individuals in this group did use VCT (excluded from this table)

### VCT intention and acceptability

The counsellors did not report negative life events following their services. They reported to be very well received by the household and the community, and this is in agreement with the data showing that 76.1% (95%CI 74.9-77.2 of those who indicated their willingness (intention), i.e. 32%, accepted the services they offered (Table 2). There was no difference in acceptability by past testing exposure, i.e. whether tested or not tested in the past (Table 2). Acceptability did not differ by sex but was higher in rural compared to urban areas (83.6% vs. 70.7%; age-adjusted odds ratio (AOR) 0.5, 95%CI: 0.32-0.68). VCT intention was somewhat higher in those reporting being tested in the past vs. not tested (37.5% vs. 30.4%, AOR 1.4, 95%CI: 1.16-1.68) and tended to be higher among men than women (AOR 1.4, 95%CI: 1.16-1.63). Among those who accepted home-based VCT, 20.6% had been previously tested for HIV.

**Table 2:** Intension and acceptability of home-based VCT

Age-group			Intention^1 ^(%)	Acceptability^2 ^(%)		
				All	Ever tested	
	N				No	Yes
**15-24**	Men	1011	36.8	77.2	76.5	82.2
	Women	1440	29.7	74.6	75.8	70.6
	
	Total	2451	32.6	75.8	76.1	74.6

**25-49**	Men	1065	34.7	78.5	77.8	79.1
	Women	1496	27.8	75.0	76.4	71.7
	
	Total	2561	30.7	76.5	77.1	74.8

**15-49**	Urban	3042	30.1	70.7	84.3	81.1
	Rural	1970	34.0	83.6	70.6	70.8
	Total	5012	31.6	76.1	76.6	74.7

^1 ^Intention: the proportion who indicated interest in being visited by a counsellor;

^2^Acceptability: Proportion of those intending being tested and received their result.

### Change in exposure to testing

**Figure 1:**
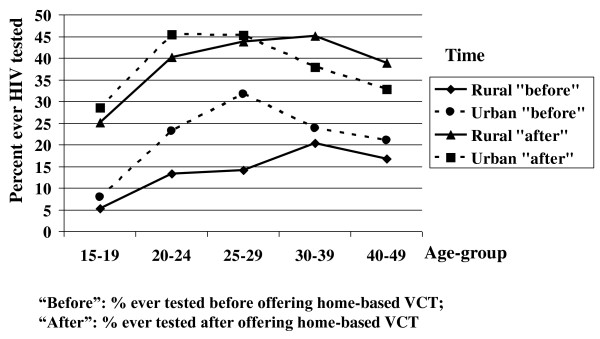
**Change in proportions ever tested for HIV comparing before with after offering home-based VCT**.

Before home-based VCT was offered, HIV testing exposure was generally low with significantly higher levels in urban than rural areas, i.e. 20.4% vs. 14.2% (AOR 1.7, 95%CI: 1.41-2.04). Exposures were particularly low in rural participants aged 15-19 years (Figure 1). After offering home-based VCT there was no difference in test rates between urban and rural areas (AOR 1), and the increase in exposure was substantial regardless of age-group.

The urban-rural stratified analyses in Table 3 show higher likelihood of being exposed to testing among the married than the unmarried and among women than men before home-based VCT was offered. After the services had been offered, these differences were not statistically significant (Table 3). In the rural areas exposures were not associated with sex and marital status in either of the two situations. Finally, the likelihood of exposure to testing tended to be biased towards the highest educated before services offered (only significant in the rural setting). The offering of home-based services appeared to have reduced this inequality as seen in the loss of significant difference between the extremes of educational levels both in urban and rural areas.

**Table 3:** Change in the distribution of ever HIV tested before versus after having implemented home-based VCT according to age-group, marital status, sex and educational attainment (n = 5012).

	Urban				Rural			
	Before		After		Before		After	
	%	AOR [95% CI]	%	AOR [95% CI]	%	AOR [95% CI]	%	AOR [ 95% CI]
**Age-group**								
15-19	7.8	1	28.6	1	5.3	1	25.1	1
20-24	23.1	2.5 [1.42-4.32]	45.4	2.0 [1.41-2.71]	13.3	2.5 [1.18-5.13]	40.2	1.9 [1.11-3.19]
25-29	31.7	3.1 [2.37-4.11]	45.2	1.8 [1.30-2.36]	14.1	2.7 [1.00-7.02]	43.7	2.1 [1.11-3.88]
30-39	23.7	1.8 [1.27-2.60]	37.8	1.2 [0.73-1.89]	20.3	4.0 [1.76-9.25]	45.1	2.1 [1.02-4.28]
40-49	21.0	1.5 [0.79-2.94]	32.6	0.9 [0.46-1.78]	16.8	3.2 [1.23-8.07]	38.8	1.6 [0.64-3.89]

**Not married**	15.3	1	35.9	1	9.5	1	32.4	1
**Ever married**	27.5	1.8 [1.24-2.62]	41.3	1.3 [0.92-1.96]	16.0	1.1 [0.64-1.73]	42.3	1.2 [0.74-1.89]

**Men**	16.8	1	37.1	1	14.3	1	42.7	1
**Women**	22.7	1.5 [1.21-1.79]	38.8	1.0 [0.74-1.40]	14.1	1.1 [0.73-1.62]	35.9	0.8 [0.66-0.92]

**Education**								
0-6 years	20.8	1	46.2	1	12.6	1	33.9	1
7 years	14.6	0.8 [0.39-1.71]	37.5	0.7 [0.48-0.91]	14.5	1.3 [0.94-1.90]	39.8	1.3 [0.96-1.73]
8-9 Years	15.2	1.0 [0.54-1.74]	36.3	0.7 [0.45-1.16]	10.5	0.9 [0.60-1.31]	39.4	1.2 [0.84-1.70]
10-11 years	17.2	0.8 [0.48-1.50]	36.9	0.7 [0.43-1.29]	14.2	1.5 [0.63-3.67]	43.7	1.8 [1.00-3.36]
≥ 12 years	31.2	1.8 [0.90-3.52]	40.3	0.7 [0.46-1.23]	29.9	1.9 [1.02-3.46]	47.0	1.3 [0.79-2.11]

## Discussion

The aim of equal access to HIV prevention, care, support and treatment is an important objective of national HIV programmes worldwide, and VCT is seen as the critical entry point in this regard. High acceptability was achieved when VCT was offered at home to all participants of a population-based survey, i.e. 76% of those expressing willingness to be tested were actually counselled and tested shortly after being offered the services as part of a population-based survey. Importantly, the home-based model of offering counselling and testing was found to have substantial effects in terms of reducing differences in HIV test rates, and this was observed as a reduction or disappearance in differences according to gender, residence and educational attainment. The findings of high acceptability reaffirm results from a previous randomised trial [12]. The trial was conducted in an urban setting, and participants were randomly allocated to VCT at the local clinic or at an optional location which was for most participants the home. Acceptance was strikingly different, i.e. 4.7 times higher uptake among the group allocated for home-based compared with clinic-based. The present findings showed comparable acceptability effects in rural settings. Similar findings have been reported in other countries in sub-Saharan Africa where offering home-based VCT has lead to increased use [11,18,19,30,31]. Health care facilities are the most frequently used location for VCT, and these findings are indicating strong acceptability barriers of clinic-based VCT and thus might be an important explanation for low HIV testing demands in sub-Saharan Africa.

Our assumption was that offering HIV testing at home would not be an attractive option for young people. However, the home-based model appeared particularly acceptable to young people as indicated by the tenfold increase in the proportion ever tested for HIV among those aged 15-19 years in rural areas (from 3% to 25%). This finding seems to agree with a recent study conducted in Zambia showing that young people ask family members for advice before seeking VCT and that disclosure is common to family members [32]. Similarly, a study in South Africa found that adolescents were ready to disclose their HIV status to family members and that they judged clinic-based VCT services to be inappropriate youth services [33]. These are indications of home-based VCT to be the youth-friendly model being searched for so long.

HIV surveys conducted in Zambia has shown higher exposures to HIV testing in urban than in rural populations [3]. In this study home-based VCT led to a marked reduction in the rural/urban differences in test rates. High acceptability was also achieved in remote areas in spite of experiencing relatively long waiting time from the pre-test counselling and HIV testing to bringing back the result, i.e. 1-2 weeks for the most remote areas vs. the next day for those in the urban setting. It is likely that this reflects the unmet need for VCT in the rural areas caused by the substantial geographical inequality in the availability still persisting. It should be noted that this striking result was achieved as part of a population survey and not as an ordinary programme. To achieve similar effects when scaling up such services is likely to depend on the extent to which capacities and resources are evenly distributed. This point was illustrated in a study in South Africa showing an accentuation of urban-rural inequalities after scaling up HIV services (including VCT services) [14].

Research indicates that gender shapes attitudes toward HIV testing in many ways, but there are no studies from high prevalence countries trying to penetrate gender differences in this regard. Our consistent finding, regardless of age and residence, was that a higher proportion of men intended to be HIV tested than women. This is consistent with the findings from a survey conducted in 1995 in the same area [3]. Before offering the home-based services, urban women appeared with 1.5 times higher likelihood of being tested than men, whereas no difference in this regard appeared in the rural setting. As an effect of higher uptake of home-based VCT, the differences disappeared in the urban area and were reversed in the rural area. This seems to be in accordance with some studies showing that women worry more about HIV and fear testing more than men [7]. A likely explanation of the sudden higher test rate among urban women in the before data is that women have been offered testing as part of prevention of mother-to-child transmission programmes. This is supported by the observation of substantially higher test rates among men than women in the mid 1990 s before such programmes had been initiated [3].

A population-based survey conducted in 1995 in the same areas as covered by the present survey revealed the likelihood of being HIV tested to be strongly associated with educational attainment, i.e. higher educated were 3 times more likely than the least educated [3]. Before our home-based services were offered, the likelihood of ever been tested also tended to be biased towards the highest educated, but the differences were reduced after the intervention. Many studies in sub-Saharan Africa have shown that HIV infections were more common among individuals with higher levels of educational attainment. However more recent data suggest that this pattern has changed and new infections are concentrating among less educated individuals [29,34,35]. In Zambia this has been observed in young adults, in whom differential survival according to level of education is unlikely, suggesting that these trends may reflect HIV incidence patterns and behaviour change [26,29], i.e. stable HIV prevalence among less educated whereas marked declines among higher educated. This evidence supports a strategy of putting high priority on preventive efforts to reaching the least educated and poor. The observed indications that home-based VCT is reducing inequalities suggest that this model could be an important part of a HIV preventive package given that strong focus is being kept on preventive counselling. From a preventive perspective, concerns have been raised related to routine testing, particularly due to the limited emphasis placed on counselling [22]. Findings from a prospective cohort study in Zimbabwe of very serious unintended increased risk taking following receipt of a negative test result might be seen as a particular warning sign with regards to effects of lost focus on preventive counselling [1].

## Conclusion

In summary, this alternative strategy of offering VCT was confirmed to be highly acceptable also in rural settings. Moreover, the home-based strategy appeared to substantially reduce existing inequalities in access. The consistency of findings of exceptionally high acceptability in other high prevalence countries indicates high level of generalization in the context of southern Africa. However, to what extent communities are accepting this home-based model might differ from a situation whereby it is offered as part of survey compared with the situation when these services are scaled up. Large-scale implementation of home-based VCT models might thus be premature, and there is an urgent need for further research efforts to examine the feasibility, acceptability, preventive effects, cost-effectiveness and negative life events of home-based VCT in community randomised trials.

## Competing interests

The authors declare that they have no competing interests.

## Authors' contributions

All authors contributed to the paper. WM took part in the analysis and writing of paper drafts. MJ took part in the analysis and parts of the draft writing. CM and KF were involved in initiating the survey, in the data collection, analysis of data and paper writing. All authors reviewed the final draft of the manuscript.

## Pre-publication history

The pre-publication history for this paper can be accessed here:

http://www.biomedcentral.com/1471-2458/10/347/prepub
